# The impact of COVID‐19 on hay fever treatment in Japan: A retrospective cohort study based on the Japanese claims database

**DOI:** 10.1002/clt2.12394

**Published:** 2024-09-17

**Authors:** Yasutsugu Akasaki, Takenori Inomata, Masao Iwagami, Jaemyoung Sung, Ken Nagino, Takeya Adachi, Hideaki Morita, Mayumi Tamari, Keigo Kainuma, Keiko Kan‐o, Hiroaki Ogata, Masafumi Sakashita, Masaki Futamura, Yosuke Kurashima, Saeko Nakajima, Katsunori Masaki, Yasushi Ogawa, Sakura Sato, Akihiro Miyagawa, Akie Midorikawa‐Inomata, Keiichi Fujimoto, Yuichi Okumura, Kenta Fujio, Tianxiang Huang, Kunihiko Hirosawa, Yuki Morooka, Akira Murakami, Shintaro Nakao

**Affiliations:** ^1^ Department of Ophthalmology Juntendo University Graduate School of Medicine Tokyo Japan; ^2^ Department of Digital Medicine Juntendo University Graduate School of Medicine Tokyo Japan; ^3^ ENGAGE‐Task Force Tokyo Japan; ^4^ Department of Hospital Administration Juntendo University Graduate School of Medicine Tokyo Japan; ^5^ Department of Telemedicine and Mobile Health Juntendo University Graduate School of Medicine Tokyo Japan; ^6^ AI Incubation Farm Juntendo University Graduate School of Medicine Tokyo Japan; ^7^ Department of Health Services Research Institute of Medicine University of Tsukuba Ibaraki Japan; ^8^ Department of Dermatology Keio University School of Medicine Tokyo Japan; ^9^ Department of Medical Regulatory Science Graduate School of Medical Science Kyoto Prefectural University of Medicine Kyoto Japan; ^10^ Department of Allergy and Clinical Immunology National Research Institute for Child Health and Development Tokyo Japan; ^11^ Allergy Center National Center for Child Health and Development Tokyo Japan; ^12^ Division of Molecular Genetics Research Center for Medical Science The Jikei University School of Medicine Tokyo Japan; ^13^ Mie National Hospital Mie Japan; ^14^ Department of Respiratory Medicine Graduate School of Medical Sciences Kyushu University Fukuoka Japan; ^15^ Division of Otorhinolaryngology Head and Neck Surgery Department of Sensory and Locomotor Medicine University of Fukui Fukui Japan; ^16^ Department of Pediatrics National Hospital Organization Nagoya Medical Center Aichi Japan; ^17^ Department of Innovative Medicine Graduate School of Medicine Chiba University Chiba Japan; ^18^ Institute for Advanced Academic Research Chiba University Chiba Japan; ^19^ Future Mucosal Vaccine Research and Development Synergy Institute Chiba University Chiba Japan; ^20^ Department of Pathology/Medicine Allergy and Vaccines CU‐UCSD Center for Mucosal Immunology University of California San Diego California USA; ^21^ Mucosal Immunology and Allergy Therapeutics Institute for Global Prominent Research Graduate School of Medicine Chiba University Chiba Japan; ^22^ Department of Drug Discovery for Inflammatory Skin Diseases Kyoto University Graduate School of Medicine Kyoto Japan; ^23^ Division of Pulmonary Medicine Department of Medicine Keio University School of Medicine Tokyo Japan; ^24^ Department of Advanced Medicine Nagoya University Hospital Aichi Japan; ^25^ Department of Allergy Clinical Research Center for Allergy and Rheumatology NHO Sagamihara National Hospital Kanagawa Japan

**Keywords:** allergic conjunctivitis, allergic rhinitis, COVID‐19, hay fever, SARS‐CoV‐2

## Abstract

**Background:**

Hay fever (HF) presents with various symptoms, including allergic conjunctivitis and rhinitis, and requires cross‐organ treatment. This study assessed the impact of the coronavirus disease 2019 (COVID‐19) pandemic on HF treatment trends.

**Methods:**

This retrospective cohort study utilized data from the JMDC database collected between January 2018 and May 2021. Patients with HF were identified based on the relevant International Classification of Diseases 10th Revision diagnosis codes and the prescription of HF‐related medications. The treatment approaches were compared during the cedar and cypress pollen allergy season (January to May in Japan) before and during the COVID‐19 pandemic (2018 and 2019, and 2020 and 2021, respectively).

**Results:**

This study included 2,598,178 patients with HF. The numbers of prescribed HF‐related claims in 2018, 2019, 2020, and 2021 were 3,332,854, 3,534,198, 2,774,380, and 2,786,681 times, respectively. Oral second‐generation antihistamine prescriptions decreased by >10% from 2019 to 2020, with a <10% change in the subsequent year. Anti‐allergic eye drop prescriptions also decreased by >10% from 2019 to 2020 but increased by >10% from 2020 to 2021. Compared with 2018, 2019, and 2020, the number of claims in the rhinitis symptoms dominant group was significantly decreased in 2021 (*p* < 0.001, all). In contrast, the number of claims in the eye symptoms dominant group and the rhinitis and eye symptoms dominant group increased in 2021 compared with that in 2018, 2019, and 2020 (*p* < 0.001, all).

**Conclusion:**

Changes in HF treatment and related outcomes could be attributed to lifestyle modifications resulting from the COVID‐19 pandemic. Measures, such as limiting outdoor activities and adopting mask‐wearing practices may have influenced HF symptoms, preventive behaviors, and the overall approach to treating HF.

## 1 INTRODUCTION

Since the first reported case of coronavirus disease 2019 (COVID‐19) in December 2019,^1^ its rapid and global spread has profoundly impacted the lifestyle and environment of the general population.^2^ To limit transmission, several countries implemented stringent measures, such as social distancing and mandatory mask‐wearing policies,^3^, ^4^ which heightened public awareness of hygiene practices,^5^, ^6^, ^7^ leading to a decline in the incidence of various non‐COVID‐19 infectious and respiratory diseases.^8^, ^9^, ^10^ Notably, substantial reductions in global influenza infections^8^, ^9^ and hospitalizations related to asthma were reported,^10^ indicating secondary effects of pandemic‐related policies. However, concerns have been raised regarding the exacerbation of conditions requiring regular follow‐ups and close monitoring.^11^

Hay fever (HF), characterized by allergic rhinitis and conjunctivitis,^12^, ^13^, ^14^ is an immune‐mediated systemic disorder that necessitates personalized treatment approaches.^13^, ^15^, ^16^ The care‐seeking behavior and treatment of HF may have been influenced by lifestyle and behavioral changes that occurred during the COVID‐19 pandemic. Previous studies^17^, ^18^, ^19^, ^20^ have reported that the widespread adoption of mask‐wearing and restrictions on outdoor activities have potentially served as preventive measures for HF. Moreover, our previous study revealed changes in HF care‐seeking patterns during the COVID‐19 pandemic using a claims database in Japan.^21^ The study identified a substantial reduction in outpatient visits for HF during the COVID‐19 pandemic; however, changes in HF treatment during the COVID‐19 pandemic have not yet been investigated.

In this study, we evaluated changes in HF prescription medication usage and treatment patterns during the COVID‐19 pandemic using the claims database from JMDC Inc.^22^, ^23^, ^24^ Our objective was to assess the impact of the COVID‐19 pandemic on HF treatment trends.

## 2 METHODS

### 2.1 Study design, data source, and source population

This retrospective cohort study was conducted utilizing the JMDC database (JMDC Inc., Tokyo, Japan)^22^, ^23^, ^24^ between January 2018 and May 2021. Our previous study described the study design and JMDC data source, an anonymized database comprising approximately 13 million health insurance recipients aged 0–74 years as of April 2022.^21^ The JMDC includes information regarding claim type (hospitalization, outpatient treatment, drug preparation, and dental treatment), recorded diagnosis based on the International Statistical Classification of Diseases and Related Health Problems 10th revision (ICD‐10), drug codes for electronic claim processing, and dates of drug prescriptions.^23^ In the Japanese health insurance system, claims are generated per patient, medical facility visited, month, and claim type.^25^ The JMDC database, through claims data, provides counts of prescriptions for specific medications of interest and co‐prescribed medications within the same claim for selected patients.

This study was approved by the Independent Ethics Committee of the Juntendo University Faculty of Medicine (approval number: E21‐0304‐M01) and followed the principles outlined in the Declaration of Helsinki. The need for informed consent was waived owing to the anonymous nature of the JMDC database.

### 2.2 Case identification

The case identification process for individuals with continuous records during the study period was completed in our previous study according to the scheme shown in Figure online.^21^ This study assessed changes in HF prescription medication usage and treatment patterns for HF during the COVID‐19 pandemic. Our analysis was based on the data from 2,598,178 individuals (51.4% male, median age [interquartile range (IQR)]; 32 [11–46] years) identified as having had HF during the HF seasons between 2018 and 2021. The median age and age distributions of patients with HF as of January 2018 are shown in Table S1 online. The proportion of pediatric patients (≤19 years old) was significantly reduced before the COVID‐19 pandemic compared with that during the COVID‐19 pandemic (39.9% vs. 39.5%, *p* < 0.001, χ^2^ test). Additional information regarding the characteristics of patients with HF during the HF and non‐HF seasons is provided in Table S2 online.

Here, we briefly describe the case identification process used in this study, which only included individuals with continuous records throughout the study period. The case identification process for the included patients was conducted as follows:

1. Patients with HF:
  We extracted the ICD‐10 codes for HF‐related diseases (Table S3 online) and prescription codes for HF‐related medications (Table S4 online). These were selected by the Empowering Next Generation Allergist/Immunologist toward Global Excellence (ENGAGE) Task Force toward 2030, which consists of nominees selected by seven allergy‐related societies in Japan.^26^ HF‐related claims were defined as monthly insurance claims for HF‐related medications. We classified patients as having HF based on the diagnosis and the prescription of related medications during an outpatient encounter for HF. To determine the medications prescribed, we used the data included in the outpatient treatment claims or drug preparations used, including abortive, oral, topical, and injection (intravenous, subcutaneous, intramuscular, or other) types of preparations. We classified HF‐related medications into 26 types based on their mechanisms of action and administration routes. We included the HF‐related medications prescribed after the patient was given ICD‐10 codes for HF‐related diseases in the analysis.
2. Patients with HF during HF seasons:
  We defined the period from January to May as the HF season, as this is when cedar and cypress pollen‐related allergies increase in prevalence in Japan due to increased dispersion.^13^, ^27^

### 2.3 Treatment patterns for allergic conjunctivitis

We defined “combinations of medications” as drugs prescribed within the same claims. The combinations of medications used for allergic conjunctivitis treatment were classified into five groups (Table S5 online) based on the guidelines for allergic conjunctival diseases.^28^

### 2.4 Symptom groups

The combinations of medications were classified into three symptom groups (eye symptoms dominant, rhinitis and eye symptoms dominant, and rhinitis symptoms dominant groups) based on the symptoms derived from the main prescribed HF‐related disease medications.

### 2.5 Treatment patterns for allergic rhinitis

We classified the combinations of medications used for allergic rhinitis into five groups (Table S6 online) based on the guidelines for allergic rhinitis management in Japan.^29^ Groups I–III reflected claims with presumed symptom severity in increasing order from mild to severe, and Group IV reflected those who received other combinations of medications, including second‐generation antihistamines and nasal steroid drops. Group V included claims without prescription medications for allergic rhinitis.

### 2.6 Follow‐up survey for regular outpatient visits

A follow‐up survey was conducted to determine whether the group of patients with HF who had visited as outpatients before the COVID‐19 pandemic had continuously visited as outpatients during the COVID‐19 pandemic.

### 2.7 Statistical analysis

We compared the characteristics of patients with HF during the HF and non‐HF seasons. Continuous variables were presented as the mean and standard deviation or median and IQR. We performed Mann–Whitney *U* tests for non‐normally distributed continuous variables based on the results of the Shapiro–Wilk tests. Categorical variables were presented as percentages and analyzed using χ^2^ tests.

We conducted a comparative analysis of prescriptions of HF‐related medications and claims during the COVID‐19 pandemic. We examined the number of claims and presented them as counts and compared the number of claims during HF seasons before and during the pandemic (2018 and 2019 as well as 2020 and 2021, respectively) using a *t*‐test. We also examined the number of prescriptions for each HF‐related medication during the HF season and presented them as counts.

Furthermore, we presented and compared the annual percentage change in the number of prescriptions for each HF‐related medication during the HF season and analyzed the treatment patterns for allergic conjunctivitis and rhinitis during the HF season by categorizing patients into groups I–V, and each group's claims were presented as counts and percentages.

Finally, as a follow‐up survey for regular outpatient visits, we calculated the number of patients with HF who visited as outpatients during the 2018 and 2019 HF seasons. Among these patients with HF, we calculated the number of patients who had continuously visited as outpatients during the 2020 and 2021 HF seasons. We also calculated the number of HF‐related drugs prescribed for patients with HF for each year from 2018 to 2021 and performed all statistical analyses using STATA/MP version 16.1 (Stata Corp., Texas, USA) and GraphPad Prism version 9.1.2 (GraphPad Software, California, USA). *p* < 0.05 was considered statistically significant.

## 3 RESULTS

### 3.1 Trends in the number of claims for HF‐related medications during COVID‐19

**FIGURE 1:**
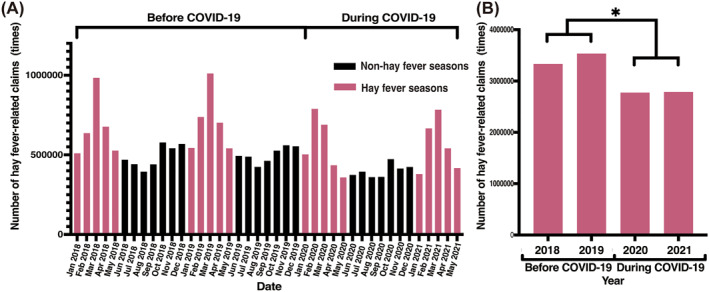
Changes during the COVID‐19 pandemic for each item per year. (A) Monthly hay fever‐related claims between January 2018 and May 2021. (B) Total hay fever‐related claims during the hay fever season between 2018 and 2021 (*p = 0.023 [*t*‐test]). COVID‐19, coronavirus disease 2019.

The total number of HF‐related claims between January 2018 and May 2021 was 12,428,113 (Figure 1A). During the HF season, the total number of claims in 2018, 2019, 2020, and 2021 was 3,332,854, 3,534,198, 2,774,380, and 2,786,681, respectively, indicating a decreasing pattern during the COVID‐19 pandemic (Figure 1B, *p = 0.023, *t*‐test).

### 3.2 Change in the number of prescriptions for HF‐related medications during COVID‐19

Table 1 lists the total number of prescriptions for each medication during the HF season from 2018 to 2021. Oral second‐generation antihistamines were the most prescribed medication, accounting for 43.7%–45.0% of all prescriptions. Anti‐allergic eye drops (ED) were the most prescribed ophthalmic medications, and steroid nasal drops (ND) were the most prescribed ND. Figure S2 online depicts the total number of prescriptions for each medication during the HF season from 2018 to 2021.

**TABLE 1:** Prescriptions of hay fever‐related medications during hay fever season.

COVID‐19	Before COVID‐19		During COVID‐19		Total
Year	2018	2019	2020	2021	
Medicine					
Eye drops					
Antiallergic	760,531	830,694	615,742	761,003	2,967,970
Steroid	289,198	297,193	220,458	254,791	1,061,640
NSAID	28,083	29,358	27,532	29,484	114,457
Immunosuppressive	2223	2356	1970	2435	8984
Eye ointments					
Steroid	71,381	77,508	66,048	75,509	290,446
Oral medications					
2nd‐generation antihistamine	2,710,900	2,907,725	2,272,832	2,299,100	10,190,557
LT receptor antagonist	694,182	748,235	565,594	515,324	2,523,335
1st‐generation antihistamine	386,172	335,699	181,635	122,548	1,026,054
Steroid	134,647	151,079	130,284	134,718	550,728
Steroid + 1st‐generation antihistamine	155,938	160,269	108,185	108,296	532,688
Kampo^a^	60,120	63,929	44,475	37,954	206,478
2nd‐generation antihistamine + *α*‐stimulator	52,963	53,712	36,234	36,629	179,538
Chemical mediator release inhibitor^b^	26,339	26,359	20,367	20,063	93,128
Th2 cytokine inhibitor^c^	15,437	16,319	13,352	12,539	57,647
PG	10,495	10,400	7427	9511	37,833
Patch medicines^d^					
2nd‐generation antihistamine	98	3472	4252	4388	12,212
Nasal drops					
Steroid	571,722	637,785	489,551	571,596	2,270,654
Vasoconstrictor	74,885	76,423	53,062	52,773	257,103
Antihistamine	14,404	14,478	10,259	11,518	50,659
Chemical mediator release inhibitor^b^	6168	6169	4564	5521	22,422
Injections					
Steroid	65,205	76,539	74,893	86,865	303,502
Non‐specific allassotherapy^e^	39,416	41,371	38,252	39,955	158,994
Antihistamine	4941	5393	5116	5927	21,377
Anti‐IgE Ab	575	664	913	1447	3599
Other					
Allergen immunotherapy	20,611	40,225	52,094	61,933	174,863
Inhalants	1138	1058	791	588	3575

Abbreviations: COVID‐19, coronavirus disease 2019; NSAIDs, non‐steroidal anti‐inflammatory drugs, LT, leukotriene; PG, prostaglandin D2 receptor/thromboxane A2 receptor antagonist.

^a^ Kampo: Sho‐seiryu‐to (kampo) inhibits the production and release of chemical mediators, demonstrating clinical efficacy for managing allergic rhinitis^29^, ^30^.

^b^ Chemical mediator release inhibitor: These inhibitors suppress chemical mediator release from mast cells; synonymous with mast cell stabilizer^29^, ^31^.

^c^ Th2 cytokine inhibitors: They suppress the production of Th2 cytokines to reduce allergic inflammation^29^, ^32^.

^d^ Patch medicines: Second‐generation antihistamine patch medicines were developed in Japan as the transdermal drug delivery system for allergic rhinitis and their efficacy and safety had been assessed^33^.

^e^ Non‐specific allassotherapy: An extract from inflammatory rabbit skin, inoculated with vaccinia virus or histamine dihydrochloride, along with human normal immunoglobulin is used to treat allergic rhinitis^29^, ^34^, ^35^.

**FIGURE 2:**
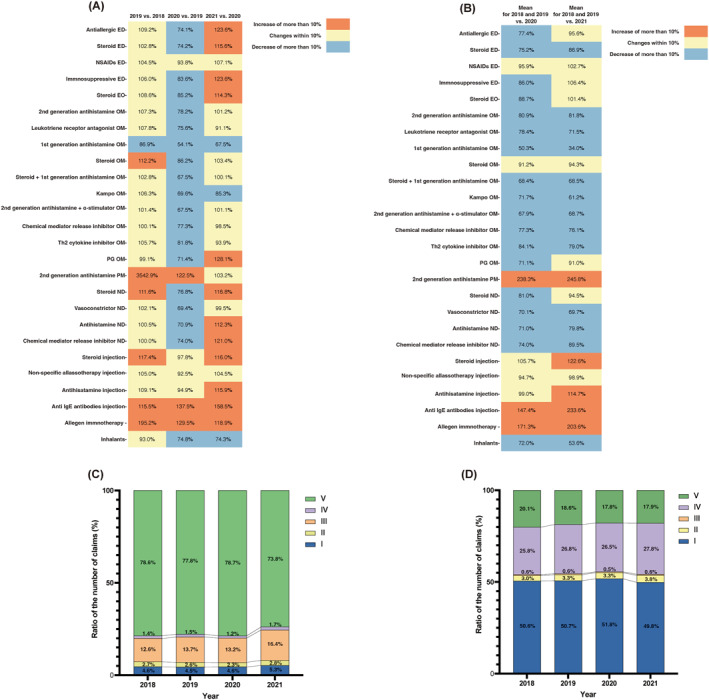
Changes in the number of prescriptions and treatment patterns for allergic conjunctivitis and allergic rhinitis during the hay fever season each year. (A) Changes in the number of prescriptions for each medication class (2019 vs. 2018, 2020 vs. 2019, and 2021 vs. 2020). (B) Mean change in the number of prescriptions for each medication (2020 vs. 2018 and 2019, 2021 vs. 2018 and 2019). Each percentage change is color‐coded based on its value (increase of >10%: red, within 10%: yellow, decrease of >10%: blue). (C) The ratio of each treatment pattern group's claims to the total number of claims by year for allergic conjunctivitis: Group I: anti‐allergic eye drops; Group II: anti‐allergic eye drops + steroid eye drops or NSAID eye drops; Group III: anti‐allergic eye drops + anti‐allergic oral or patch medicine; Group IV: anti‐allergic eye drops + anti‐allergic oral or patch medicines + steroid eye drops or NSAID eye drops; Group V: not prescribed anti‐allergic eye drops. (D) The ratio of each treatment pattern group's claims to the total number of claims by year for allergic rhinitis: Group I: mild; Group II: moderate; Group III: severe; Group IV: other combinations; Group V: not prescribed allergic rhinitis‐related medications. ED, eye drops; NSAIDs, non‐steroidal anti‐inflammatory drugs; EO, eye ointments; OM, oral medicines; PG, prostaglandin D2 receptor/thromboxane A2 receptor antagonist; ND, nasal drops; PM, patch medicines.

Figure 2 presents the year‐by‐year percentage change (Figure 2A) and compares the number of prescriptions for each medication during COVID‐19 (Figure 2B). Oral second‐generation antihistamines increased by 7.3% between 2018 and 2019, followed by a 21.8% decrease between 2019 and 2020 and a 1.2% increase in 2021 compared with 2020. In comparison, anti‐allergic ED increased by 9.2% in 2018 and 2019, decreased by 25.9% in 2020 compared with that in 2019, and increased by 23.6% in 2021 compared with that in 2020. Steroid ND increased by 11.6% in 2019 compared with that in 2018, decreased by 23.2% in 2020 compared with that in 2019, and increased by more than 16.8% in 2021 compared with that in 2020 (Figure 2A).

Regarding overall changes, 19 out of 26 medications showed a change of 10% or less in the number of prescriptions between 2018 and 2019. However, in 2020, 19 medications showed a decrease of 10% or more compared with 2019. In 2021, the number of prescriptions for 12 medications increased by >10% compared with that in 2020, and 11 medications showed a change of 10% or less.

Figure 2B compares the number of prescriptions during COVID‐19. Second‐generation antihistamines decreased by 19.1% in 2020 compared with that in 2018 and 2019, and a further decrease of 18.2% was observed in 2021. Anti‐allergic ED showed a 22.6% decrease in 2020 compared with that in 2018 and 2019, but only a 4.4% decrease in 2021. Steroid ND exhibited a 19.0% decrease in 2020 compared with that in 2018 and 2019, and a 5.5% decrease in 2021.

### 3.3 Change in the number of claims based on symptom group

Table 2 lists the number of claims based on the symptom groups. The number of claims in the rhinitis symptoms dominant group was more than two‐thirds throughout the period and significantly decreased in 2021 compared with that in 2018, 2019, and 2020 (*p* < 0.001, all). The number of claims in the eye symptoms dominant group increased in 2021 compared with that in 2018, 2019, and 2020 (*p* < 0.001, all). The number of claims in the rhinitis and eye symptoms dominant group increased in 2021 compared with that in 2018, 2019, and 2020 (*p* < 0.001, all).

**TABLE 2:** Number of claims based on symptom group.

Patients	Patients with HF during the HF seasons before the COVID‐19 pandemic		Patients with HF during the HF seasons during the COVID‐19 pandemic		Total
Year	2018	2019	2020	2021	
Symptom group					
Eye symptoms dominant group	245,192 (7.4%)	248,403 (7.0%)	191,227 (6.9%)	225,310 (8.1%)	910,132 (7.3%)
Rhinitis and eye symptoms dominant group	468,200 (14.0%)	537,651 (15.2%)	400,917 (14.5%)	503,875 (18.1%)	1,910,643 (15.4%)
Rhinitis symptoms dominant group	2,619,462 (78.6%)	2,748,144 (77.8%)	2,182,236 (78.7%)	2,057,496 (73.8%)	9,607,338 (77.3%)
Total	3,332,854 (100%)	3,534,198 (100%)	2,774,380 (100%)	2,786,681 (100%)	12,428,113 (100%)

*Note*: Eye symptoms dominant group: Prescribed anti‐allergic ED but not anti‐allergic OM or PM (Group I + Group II); Rhinitis and eye symptoms dominant group: Prescribed anti‐allergic ED and anti‐allergic OM or PM (Group III + Group IV); Rhinitis symptoms dominant group: Not prescribed anti‐allergic ED (Group V).

Abbreviations: COVID‐19, coronavirus disease 2019; ED, eye drops; HF, hay fever; OM, oral medicines; PM, patch medicines.

### 3.4 Changes in treatment patterns for allergic conjunctivitis and allergic rhinitis during COVID‐19

Table 3 lists the annual claims for allergic conjunctivitis by treatment group during the HF season and their ratio to total annual claims. Figure 2C illustrates the changing annual claim ratios for each group. The per‐group claim ratios in 2020 remained within 1% compared to 2018 and 2019; in 2021, the percentage of claims notably increased in Groups I–IV compared with that in the previous three years. Additionally, in Group III, the number of claims in 2021 was approximately 3% higher than that in the previous three years.

**TABLE 3:** Number of claims for allergic conjunctivitis treatment.

COVID‐19	Before COVID‐19		During COVID‐19		Total
Year	2018	2019	2020	2021	
Group					
I	154,132 (4.6%)	157,483 (4.5%)	126,565 (4.6%)	148,440 (5.3%)	586,620
II	91,060 (2.7%)	90,920 (2.6%)	64,662 (2.3%)	76,870 (2.8%)	323,512
III	420,844 (12.6%)	484,292 (13.7%)	367,277 (13.2%)	457,870 (16.4%)	1,730,283
IV	47,356 (1.4%)	53,359 (1.5%)	33,640 (1.2%)	46,005 (1.7%)	180,360
V	2,619,462 (78.6%)	2,748,144 (77.8%)	2,182,236 (78.7%)	2,057,496 (73.8%)	9,607,338
Total	3,332,854 (100%)	3,534,198 (100%)	2,774,380 (100%)	2,786,681 (100%)	12,428,113

*Note*: I, Anti‐allergic eye drops; II, Anti‐allergic eye drops + steroid eye drops or NSAID eye drops; III, Anti‐allergic eye drops + anti‐allergic oral or patch medicines; IV, Anti‐allergic eye drops + anti‐allergic oral or patch medicine + steroid eye drops or NSAID eye drops; V, No prescribed anti‐allergic eye drops.

Abbreviations: COVID‐19, coronavirus disease; NSAID, non‐steroidal anti‐inflammatory drug.

Table 4 lists the annual claims for allergic rhinitis by treatment group during the HF season and their ratio to the annual total. Figure 2D illustrates the changing ratios of each group throughout the 4‐year period. Unlike allergic conjunctivitis, no consistent trend was observed between the groups.

**TABLE 4:** Number of claims for allergic rhinitis treatment.

COVID‐19	Before COVID‐19		During COVID‐19		Total
Year	2018	2019	2020	2021	
Group					
I	1,685,044 (50.6%)	1,792,354 (50.7%)	1,437,213 (51.8%)	1,387,943 (49.8%)	6,302,554
II	98,640 (3.0%)	115,019 (3.3%)	92,165 (3.3%)	106,550 (3.8%)	412,374
III	19,605 (0.6%)	21,292 (0.6%)	14,445 (0.5%)	17,442 (0.6%)	72,784
IV	861,092 (25.8%)	948,120 (26.8%)	735,916 (26.5%)	776,045 (27.8%)	3,321,173
V	668,473 (20.1%)	657,413 (18.6%)	494,641 (17.8%)	498,701 (17.9%)	2,319,228
Total	3,332,854 (100%)	3,534,198 (100%)	2,774,380 (100%)	2,786,681 (100%)	12,428,113

*Note*: I, mild; II, moderate; III, severe; IV, other combinations; V, not prescribed allergic rhinitis medications.

Abbreviations: COVID‐19, coronavirus disease 2019.

### 3.5 Follow‐up survey for outpatient visits among patients with HF before the COVID‐19 pandemic

Table S7 online shows that 1,084,620 individuals visited as outpatients during the 2018 and 2019 HF seasons, of which 746,932 visited as outpatients during the 2020 HF season and 719,657 visited as outpatients during the 2021 HF season. The number of prescriptions for HF‐related medications was 4,977,468 in 2018, 5,027,852 in 2019, 3,356,985 in 2020, and 3,325,623 in 2021.

## 4 DISCUSSION

To elucidate the impact of the COVID‐19 pandemic on HF‐related healthcare patterns, we analyzed HF treatment trends during the pandemic using a Japanese claims database. Our results indicated that HF‐related claims decreased by approximately 20% compared with pre‐pandemic numbers. Due to lifestyle changes during the pandemic, including increased mask usage, minimal out‐of‐home activities, and a potential shift in individuals' behavior or attitude toward visiting hospitals/clinics, along with decreased pollen dispersal levels, we observed a notable difference in treatment trends. Our results demonstrate a trend toward an increasing prevalence of allergic conjunctivitis, likely due to the limited impact of mask use on pollen exposure to the eye. These results offer a comprehensive understanding of post‐pandemic HF epidemiology, outpatient visits, and treatment trends, which could help determine future measures to meet changing medical needs.

Our results show that claims during HF seasons significantly decreased compared with pre‐pandemic data. Our previous study reported that HF‐related outpatient visits decreased during the COVID‐19 pandemic,^21^ and reports on influenza,^9^ allergic rhinitis,^36^ asthma,^36^ viral and allergic conjunctivitis,^37^ diabetes,^11^ and human immunodeficiency virus^38^ visits were similarly affected by the pandemic. These effects stem from efforts to minimize SARS‐CoV‐2 exposure, including avoiding hospital visits,^11^, ^38^ government enforcement of mask usage, strict hand hygiene, and limited out‐of‐home activities.^9^, ^21^, ^36^, ^37^ A previous Polish study reported an increase in self‐medication in patients who did not show such behaviors prior to the lockdown phase.^39^ In Japan, sales of over‐the‐counter (OTC) medications associated with allergic rhinitis decreased in 2020 compared with that in 2019.^40^ Considering the simultaneous drop in prescription counts and OTC medication sales during the pandemic, it appears unlikely that the decreases in outpatient visits and prescriptions for HF were due to available OTC remedies. In addition, the cedar and cypress pollen dispersal levels in spring 2020 tended to be lower than those in 2018 and 2019 throughout Japan (Figure S3 online).^41^ The trends observed in this study are likely the result of a combination of lifestyle changes, including wearing masks and limited out‐of‐home activities, and decreased pollen dispersal levels that suppress certain HF symptoms.

Using prescription data, we inferred the symptom trends of allergic conjunctivitis as a result of post‐pandemic changes. Throughout the research period, the most prescribed medication class for allergic conjunctivitis with underlying HF was anti‐allergic ED.^28^ The second most prescribed medication class was steroid ED, which are frequently used as add‐on agents for anti‐allergic ED. Both anti‐allergic and steroid drops showed a decreased prescription rate in 2020 compared with the pre‐pandemic data. These findings likely reflect the limited healthcare access and nationwide encouragement to stay at home.^19^ When comparing the two post‐pandemic years, the prescription counts for these drug classes rebounded during 2021. This increasing trend could be attributed to reduced restrictions, leading to increased pollen exposure. As a result, the eyes may have been subjected to higher pollen exposure^17^, ^42^ compared with the respiratory organs, which were protected by masks. The “2021 lifestyle,” which entailed continued mask use and reduced restrictions, likely brought out allergic conjunctivitis symptoms compared with the years with stricter restrictions.

Similarly, prescription changes for allergic rhinitis were also reviewed. The most frequently prescribed medication class was second‐generation oral antihistamines. The most frequently prescribed intranasal medication was an intranasal steroid spray. Second‐generation oral antihistamines showed a decrease in prescriptions after the start of the pandemic (2020), which persisted the following year. This is likely due to lifestyle changes, such as mask usage and decreased outdoor activity, which are known to alleviate allergic rhinitis.^17^, ^18^, ^19^, ^20^, ^36^ Similarly, intranasal steroid sprays showed an overall decrease in prescription counts during the pandemic period. However, we observed an increase in steroid spray prescriptions in 2021 compared with 2020. The divergent trend between the two medications may be due to the limited effects of oral antihistamines on nasal congestion,^43^ which motivated patients to seek locally acting remedies.^44^ Notably, allergen immunotherapy showed a constant increase, reflecting the increasing demand for desensitization therapies.^45^ In line with previous reports, first‐generation antihistamines have decreased, likely owing to their short‐acting nature and side effect profiles, including central nervous system depression and anticholinergic effects.^46^, ^47^ Overall, patients may experience improved symptoms due to lifestyle changes brought on by the pandemic; however, patients with severe or local symptoms may return to receive care.

Furthermore, this study compared the ocular and nasal symptom trends of allergic conjunctivitis and rhinitis. When comparing the pre‐ and post‐pandemic prescription counts, we observed a decrease in the number of prescriptions for both allergic conjunctivitis and rhinitis. However, prescriptions for allergic rhinitis, inferred from prescribed second‐generation oral antihistamines, remained stable during the pandemic years at a 101.2% year‐on‐year change. Allergic conjunctivitis notably increased during the latter year, at a 123.6% year‐on‐year change when looking at anti‐allergic eye drop prescriptions. These results may indicate a relative increase in the demand for outpatient care for allergic conjunctivitis compared with rhinitis. The contrasting observations seen in the two related diseases appear to stem from the current conventional use of masks, which may suppress nasal symptoms but not conjunctivitis.^17^

Regarding the disease severity trends surrounding the pandemic, we did not observe a significant ratio shift in the treatment groups for allergic rhinitis. However, in 2021, we noted an increase in all medicated groups for allergic conjunctivitis (eye symptoms dominant group and rhinitis and eye symptoms dominant group) compared with the previous three years. This may suggest a comparative increase in medication‐controlled allergic conjunctivitis in patients receiving HF care in 2021. One underlying reason could be the fear of SARS‐CoV‐2 spreading through the ocular surface,^48^ leading to a widespread aversion to eye rubbing and motivating patients to medicate ocular symptoms in 2021. Another reason may be environmental predisposition and regulatory changes that contribute to increased allergic conjunctivitis care. A report indicated increased pollen dispersion in 2021 compared with 2020.^41^ The lack of ocular protection may have exposed the eye to allergens with reduced outdoor restrictions, whereas masks provided nasal protection.^17^ While previous reports^17^, ^20^ agree on the improvement of allergic rhinitis symptoms with mask use during the pandemic, there is inconsistent evidence for conjunctivitis, with one reporting improvement^20^ and one without.^17^ To the best of our knowledge, no studies have reported worsening allergic conjunctivitis with mask use, but the directional air current toward the eyes may contribute to dry eye^49^, ^50^, ^51^ and worsen the symptoms.^52^, ^53^, ^54^ Future studies should consider environmental factors, including pollen. In a study using the Korean National Health Insurance claims database comparing examination counts for allergic rhinitis and conjunctivitis affected by the pandemic, a decreased average visit for allergic rhinitis was reported, whereas that for allergic conjunctivitis remained stable.^36^ These results also suggest a relative persistence of allergic conjunctivitis symptoms compared with rhinitis symptoms in South Korea. Lifestyle changes with long‐term mask use and hygiene are a widely observed phenomenon that calls for a population‐wide preventive and interventional strategy to accommodate the anticipated need for allergic conjunctivitis management.

This study has some limitations. First, this study used data limited to specific diagnoses and prescriptions, and information regarding other associated diseases and medications was not evaluated. Therefore, the findings may not fully capture the broader picture of the medical landscape. Second, HF‐related disease codes may be assigned for insurance purposes when prescribing HF‐related drugs in practice. Thus, the medications of interest may have been prescribed for reasons other than HF‐related diseases, such as chronic urticaria. However, it is difficult to identify the true disease or the onset time of HF because there is no test value or medical record information in the claims database. Therefore, we attempted to identify the medications prescribed for actual HF‐related diseases by focusing the analysis on the period of cedar and cypress pollinosis in Japan. However, the definition of patients with HF in this study may overestimate the number of patients with HF because the definition is considered to have high sensitivity but low specificity. Third, to comply with pandemic‐related regulations, providers may have prescribed longer prescriptions during the pandemic to reduce the need for patients to visit for refills. In addition, this study did not follow up on the symptom changes for HF during the study period. Fourth, as providers may have varying criteria for selecting and increasing treatments, the treatment groups may not have accurately reflected symptom severity. Fifth, HF‐related drugs newly covered by Japanese insurance during the study period were included in the analysis. The second‐generation antihistamine patch medicines were launched in April 2018,^33^ hence the low number of prescriptions in 2018. In addition, insurance coverage of a monoclonal anti‐IgE antibody, omalizumab, for HF started in 2020.^55^ Therefore, the increased number of prescriptions of the monoclonal anti‐IgE antibody during the COVID‐19 pandemic (2020 and 2021) compared with before the COVID‐19 pandemic (2018 and 2019) may not have been caused by the pandemic. Sixth, since the JMDC database does not provide data on the residence of patients,^23^ we could not assess the trend of prescriptions for HF medications by location in this study. Furthermore, the timing of the declaration periods for a state of emergency, which was expected to affect outdoor activities, differed among regions.

However, the impact of this factor could not be evaluated in this study because of the lack of location data. Future studies should consider additional factors, such as geography and pollen dispersion, to better specify the effects of the pandemic. Moreover, utilizing mobile health‐based methodologies may enhance the accrual of comprehensive longitudinal data, especially when wide outreach is required.^13^, ^14^, ^16^, ^56^, ^57^, ^58^, ^59^, ^60^, ^61^ Finally, this study revealed a decreased proportion of pediatric visits during the COVID‐19 pandemic compared with that before the pandemic. Although the median age remained similar before and during the COVID‐19 period, selection bias may have occurred in the age distribution in this study. Conversely, the decrease in the proportion of pediatric visits for HF from this study suggests the need for appropriate allergy interventions for pediatric patients during COVID‐19.^62^

In conclusion, this study suggests that lifestyle changes and altered perspectives brought about by the COVID‐19 pandemic, such as the use of masks and reduced out‐of‐home activities, may have impacted HF symptoms and treatment patterns. These trends observed during the pandemic could have implications for the development of appropriate population health measures for HF prevention and intervention, considering the changing medical needs of affected individuals.

## AUTHOR CONTRIBUTIONS

**Yasutsugu Akasaki**: Investigation; formal analysis; visualization; writing—original draft; writing—review & editing; software; data curation. **Takenori Inomata**: Conceptualization; methodology; software; data curation; supervision; validation; investigation; funding acquisition; project administration; visualization; writing—review & editing; writing—original draft; resources. **Masao Iwagami**: Methodology; validation; formal analysis; writing—review & editing; writing—original draft. **Jaemyoung Sung**: Writing—original draft; writing—review & editing; validation; formal analysis. **Ken Nagino**: Methodology; validation; visualization; investigation; writing—original draft; writing—review & editing; formal analysis. **Takeya Adachi**: Conceptualization; validation; writing—review & editing; writing—original draft. **Hideaki Morita**: Conceptualization; writing—review & editing; validation; writing—original draft. **Mayumi Tamari**: Writing—review & editing; writing—original draft; validation; conceptualization. **Keigo Kainuma**: Conceptualization; validation; writing—review & editing; writing—original draft. **Keiko Kan‐o**: Writing—review & editing; writing—original draft; conceptualization; validation. **Hiroaki Ogata**: Conceptualization; writing—review & editing; validation; writing—original draft. **Masafumi Sakashita**: Conceptualization; validation; writing—review & editing; writing—original draft. **Masaki Futamura**: Conceptualization; validation; writing—review & editing; writing—original draft. **Yosuke Kurashima**: Validation; conceptualization; writing—original draft; writing—review & editing. **Saeko Nakajima**: Conceptualization; validation; writing—review & editing; writing—original draft. **Katsunori Masaki**: Conceptualization; validation; writing—review & editing; writing—original draft. **Yasushi Ogawa**: Conceptualization; validation; writing—review & editing; writing—original draft. **Sakura Sato**: Conceptualization; writing—original draft; writing—review & editing; validation. **Akihiro Miyagawa**: Conceptualization; validation; writing—review & editing; writing—original draft. **Akie Midorikawa‐Inomata**: Writing—review & editing; writing—original draft; funding acquisition; validation; methodology. **Keiichi Fujimoto**: Funding acquisition; writing—original draft; writing—review & editing. **Yuichi Okumura**: Writing—original draft; writing—review & editing; data curation. **Kenta Fujio**: Writing—original draft; writing—review & editing; data curation. **Tianxiang Huang**: Writing—original draft; writing—review & editing; data curation. **Kunihiko Hirosawa**: Writing—original draft; writing—review & editing; data curation. **Yuki Morooka**: Writing—original draft; writing—review & editing; data curation. **Akira Murakami**: Writing—review & editing; writing—original draft; supervision; resources; project administration. **Shintaro Nakao**: Writing—review & editing; writing—original draft; resources; supervision.

## CONFLICT OF INTEREST STATEMENT

H.M. received a grant from GlaxoSmithKline Japan. M.T. received lecture fees from Sanofi, Astra Zeneca UCB Japan, and KYORIN Pharmaceutical Co., Ltd. K.M. received a grant from Nippon Boehringer Ingelheim Co., Ltd., TAIHO Pharmaceutical Co., Ltd., and Ping An‐Shionogi Co., Ltd. K.N., Y. Okumura, and A.M.I. received personal fees from InnoJin, Inc., outside the submitted work. T.I. reports non‐financial support from Lion Corporation and Sony Network Communications Inc., grants from Johnson & Johnson Vision Care, Inc., Yuimedi, Inc., ROHTO Pharmaceutical Co., Ltd., Kobayashi Pharmaceutical Co., Ltd., Kandenko Co., Ltd., and Fukoku Co., Ltd., personal fees from Santen Pharmaceutical Co., Ltd., InnoJin, Inc., and Ono Pharmaceutical Co., Ltd., outside the submitted work. S. Nakao reports grants from Kowa Company. Ltd., Mitsubishi Tanabe Pharma Corporation, Alcon Japan, Ltd., Santen Pharmaceutical Co., Ltd., Machida Endoscope Co., Ltd., Wakamoto Pharmaceutical Co., Ltd., Bayer Yakuhin, Ltd., Senju Pharmaceutical Co., Ltd., Nippon Boehringer Ingelheim Co., Ltd., Chugai Pharmaceutical Co., Ltd., Hoya Corporation, and Novartis Pharma K.K., outside the submitted work. The remaining authors declare no competing interests.

## Supporting information

Supporting Information S1

## Data Availability

The data that support the findings of this study are available from JMDC, Inc. Restrictions apply to the availability of these data, which were used under license for this study. Data are available from https://www.jmdc.co.jp with the permission of JMDC, Inc.
