# Supplementary material for: The impact of COVID‐19 on hay fever treatment in Japan: A retrospective cohort study based on the Japanese claims database
Source: Clin Transl Allergy. 2024 Sep 17;14(9):e12394. doi: 10.1002/clt2.12394 (PMC11406147; doi:10.1002/clt2.12394)
Supplement: Supplementary file 1 — Supporting Information S1 [file CLT2-14-e12394-s001.pdf]

Supporting information

Figures

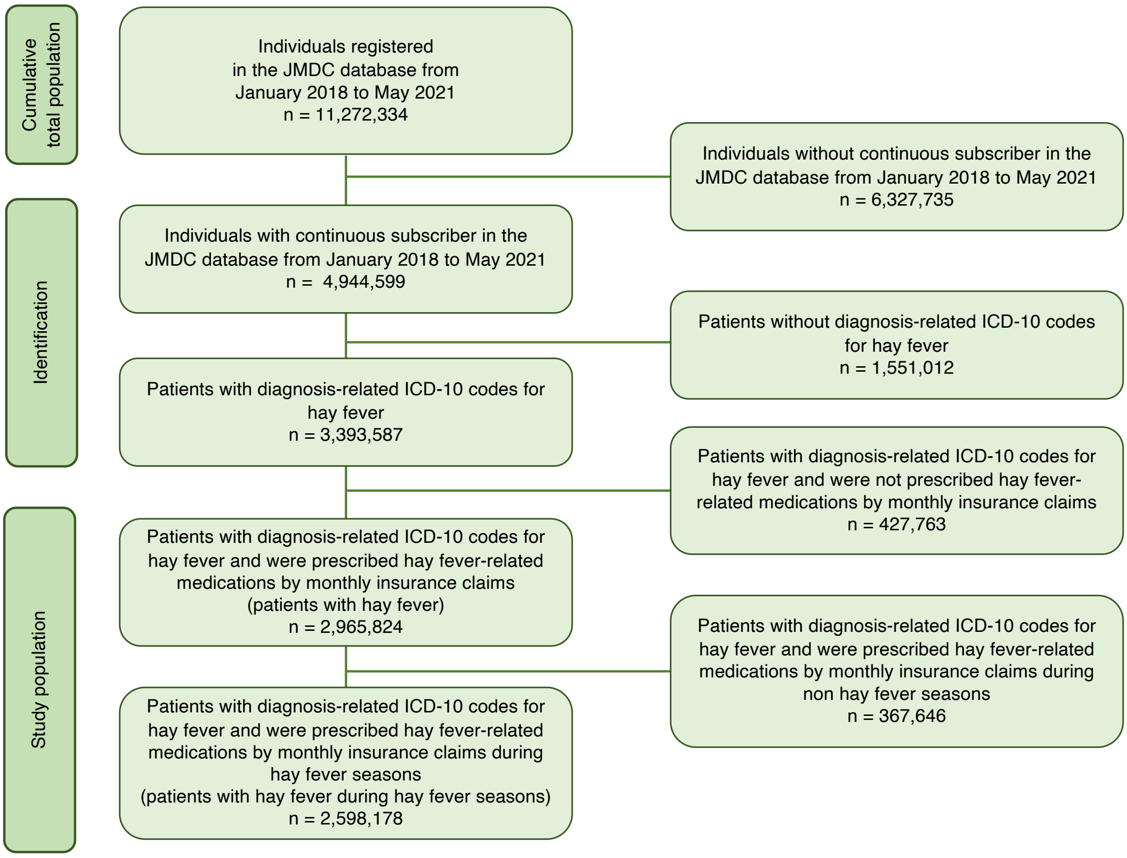

Figure S1. Case identification flowchart. Flowchart of the case identification process as reported in our previous study[1]. A total of 11,272,334 individuals were registered in the JMDC database between January 2018 and May 2021, of which 4,944,599 were identified as continuous subscribers in the JMDC database (56.3% male, mean age (± SD); 33.4 ± 18.2 years). Between January 2018 and May 2021, 2,965,824 individuals with HF were also included (51.7% male, mean age (± SD); 30.0 ± 19.0 years). Finally, 2,598,178 individuals who had HF during the HF seasons between 2018 and 2021 were identified (51.4% male, mean age (± SD); 29.6 ± 19.2 years). ICD-10, International Statistical Classification of Diseases and Related Health Problems 10th revision.

HF, hay fever

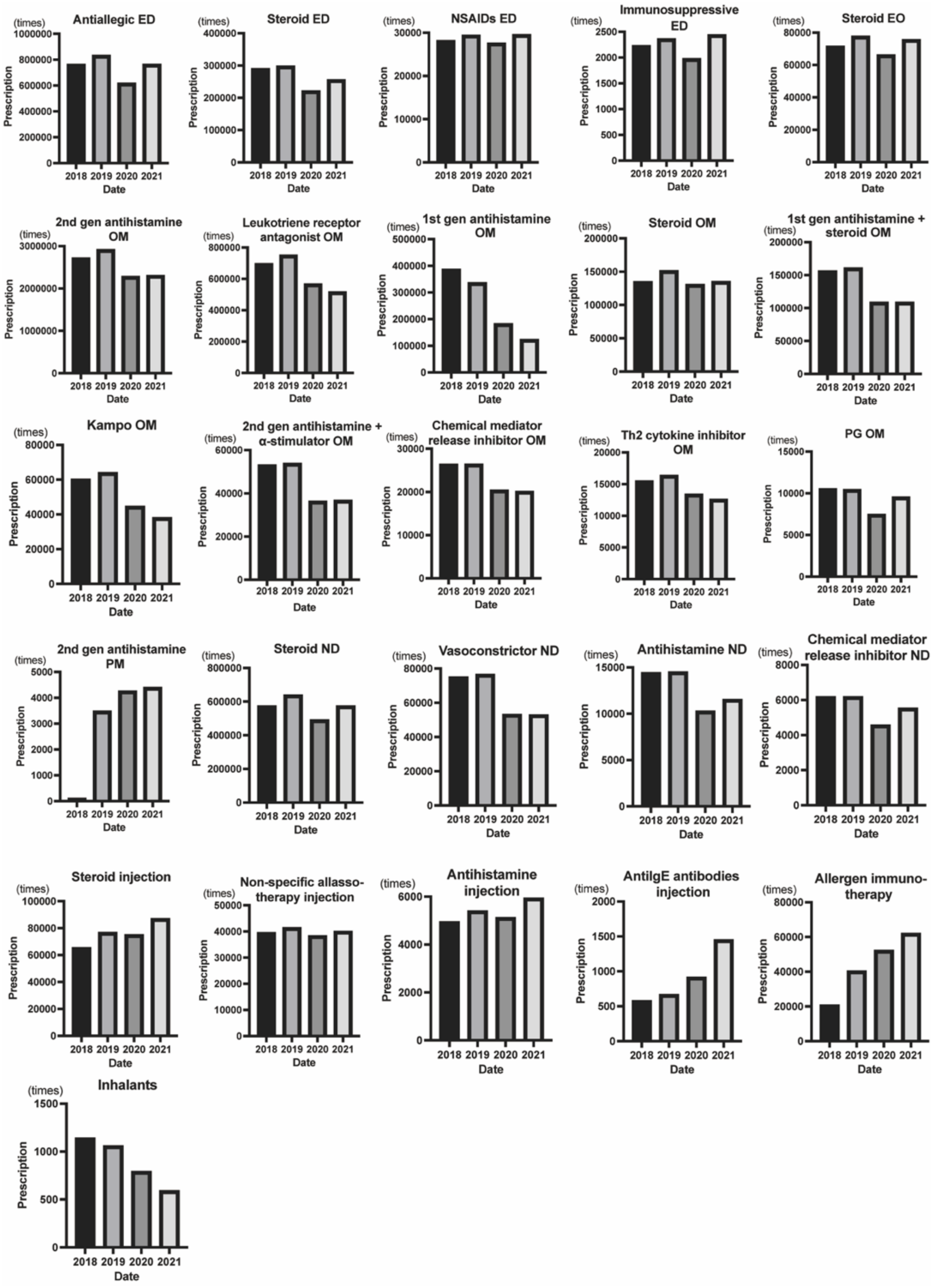

Figure S2. Total number of prescriptions written for each hay fever-related medication per year during hay fever season. n = 2,598,178. Abbreviations: ED, eye drops; NSAIDs, non-steroidal anti-inflammatory drugs; OM, oral medicine; EO, eye ointment; PG, prostaglandin D2 receptor/thromboxane A2 receptor antagonist; ND,

nasal drop; PM, patch medicine.

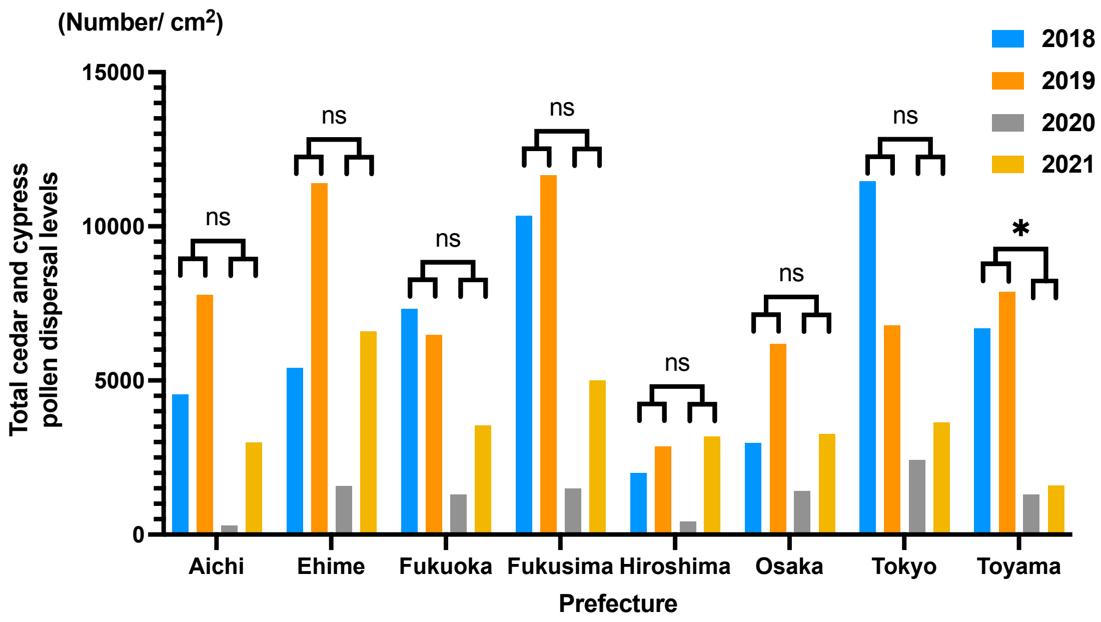

Figure S3. Total cedar and cypress pollen dispersal levels in the spring (February to April) among major prefectures in Japan. The total cedar and cypress pollen dispersal levels in spring 2020 tended to be lower than those in previous years throughout Japan. An overall trend toward lower total cedar and cypress pollen dispersal levels was observed in the spring following the COVID-19 pandemic than prior. The cedar and cypress pollen dispersal levels were significantly lower in Toyama (*P = 0.012 [*t*-test]).

Tables.

Table S1. Age distribution of patients with hay fever in this study

	**Patients with HF during the HF seasons before the COVID-19 pandemic (2018 and 2019)** **n = 2,167,796**	**Patients with HF during the HF seasons after the COVID-19 pandemic (2020 and 2021)** **n = 1,823,675**	*P*-value	**Patients with HF during the HF seasons before and after the COVID-19 pandemic (2018, 2019, 2020, and 2021)** **n = 2,598,178**
Age, years, median [IQR]	31 [10–46]	31 [9–46]	< 0.001	32 [11–46]
Age distribution group, years, number (%)			< 0.001	
0–9	515,569 (23.8%)	469,258 (25.7%)		591,237 (22.8%)
10–19	348,570 (16.1%)	251,506 (13.8%)		401,059 (15.4%)
20–29	168,184 (7.8%)	143,511 (7.9%)		218,174 (8.4%)
30–39	344,090 (15.9%)	289,403 (15.9%)		426,249 (16.4%)
40–49	403,845 (18.6%)	333,309 (18.3%)		492,227 (18.9%)
50–59	299,313 (13.8%)	257,056 (14.1%)		361,677 (13.9%)
60–69	82,112 (3.8%)	74,122 (4.1%)		100,205 (3.9%)
70+	6,113 (0.3%)	5,510 (0.3%)		7,350 (0.3%)
Total	2,167,796 (100%)	1,823,675 (100%)		2,598,178 (100%)

IQR, interquartile range; HF, hay fever; COVID-19, coronavirus disease 2019.

Table S2. Characteristics of patients during hay fever and non-hay fever seasons

Patients	Patients with hay fever during hay fever seasons n = 2,598,178	Patients with hay fever during non-hay fever seasons n = 367,646	*P*-value	Total n = 2,965,824
**Variable and category**				
Age, years, median [IQR]	32 [11–46]	35 [17–47]	< 0.001	32 [11–46]
Sex, males, number (%)	1,336,076 (51.4)	198,226 (53.9)	< 0.001	1,534,302 (51.7)

IQR, interquartile range.

Table S3. List of diagnosis-related codes for hay fever

Diagnosis	International Classification of Diseases, 10th Revision (ICD-10) code
Allergic conjunctivitis	H10.1
Vernal keratoconjunctivitis	H10.1
Hay fever	J30.1
Allergic rhinitis	J30.4
Allergic sinusitis	J30.4
Seasonal allergic rhinitis	J30.2
Allergic blepharitis	H01.1
Allergic marginal blepharitis	H01.1
Allergic rhinopharyngitis	J30.4
Giant papillary conjunctivitis	H10.4
Perennial allergic rhinitis	J30.3
Allergic rhinoconjunctivitis	H10.1 J30.1
Atopic keratoconjunctivitis	H10.4
Seasonal allergic conjunctivitis	H10.1
Perennial allergic conjunctivitis	H10.1
Gramineae pollinosis	J30.1
Orchard grass pollinosis	J30.1
Japanese cedar pollinosis	J30.1
Cypress pollinosis	J30.1
Ragweed pollinosis	J30.1
Timothy Pollinosis	J30.1
Quercus serrata pollinosis	J30.1
Birch pollinosis	J30.1
Sweet vernal grass pollinosis	J30.1
Alder pollinosis	J30.1
Artemisia pollinosis	J30.1

Table S4. List of hay fever-related medications

Generic name	Class	Administration
Sodium Azulene Sulfonate	NSAIDs	eye drops
Dipotassium Glycyrrhizinate	NSAIDs	eye drops
Pranoprofen	NSAIDs	eye drops
Bromfenac Sodium Hydrate	NSAIDs	eye drops
Sodium Cromoglicate	chemical mediator release inhibitor	eye drops
Amlexanox	chemical mediator release inhibitor	eye drops
Pemirolast Potassium	chemical mediator release inhibitor	eye drops
Tranilast	chemical mediator release inhibitor	eye drops
Ibudilast	chemical mediator release inhibitor	eye drops
Acitazanolast Hydrate	chemical mediator release inhibitor	eye drops
Ketotifen Fumarate	antihistamine	eye drops
Levocabastine Hydrochloride	antihistamine	eye drops
Olopatadine Hydrochloride	antihistamine	eye drops
Epinastine Hydrochloride	antihistamine	eye drops
Dexamethasone Metasulfobenzoate Sodium	steroid	eye drops
Dexamethasone Sodium Phosphate	steroid	eye drops

Hydrocortisone Acetate	steroid	eye drops
Fluorometholone	steroid	eye drops
Betamethasone Sodium Phosphate	steroid	eye drops
Prednisolone Acetate	steroid	eye drops
Betamethasone Sodium Phosphate + Fradiomycin Sulfate	steroid + antibiotics	eye drops
Prednisolone Acetate	steroid	ointments
Dexamethasone	steroid	ointments
Fradiomycin Sulfate, Methylprednisolone	steroid + antibiotics	ointments
Betamethasone Sodium Phosphate, Fradiomycin Sulfate	steroid + antibiotics	ointments
Ciclosporin	immunosuppressive	eye drops
Tacrolimus Hydrate	immunosuppressive	eye drops
Diphenhydramine Hydrochloride	1st-generation antihistamine	oral
Promethazine Hibenzate, Promethazine	1st-generation antihistamine	oral
Promethazine Methylenedisalicylate, Promethazine Hydrochloride	1st-generation antihistamine	oral
Alimemazine Tartrate, Trimeprazine Tartrate	1st-generation antihistamine	oral
d-Chlorpheniramine Maleate	1st-generation antihistamine	oral
Chlorpheniramine Maleate	1st-generation antihistamine	oral
Cyproheptadine Hydrochloride Hydrate	1st-generation antihistamine	oral

Homochlorcyclizine Hydrochloride	1st-generation antihistamine	oral
Clemastine Fumarate	1st-generation antihistamine	oral
Ketotifen Fumarate	2nd-generation antihistamine	oral
Azelastine Hydrochloride	2nd-generation antihistamine	oral
Oxatomide	2nd-generation antihistamine	oral
Emedastine Difumarate	2nd-generation antihistamine	oral
Epinastine Hydrochloride	2nd-generation antihistamine	oral
Ebastine	2nd-generation antihistamine	oral
Cetirizine Hydrochloride	2nd-generation antihistamine	oral
Bepotastine Besilate	2nd-generation antihistamine	oral
Fexofenadine Hydrochloride	2nd-generation antihistamine	oral
Olopatadine Hydrochloride	2nd-generation antihistamine	oral
Loratadine	2nd-generation antihistamine	oral
Levocetirizine Hydrochloride	2nd-generation antihistamine	oral
Desloratadine	2nd-generation antihistamine	oral
Bilastine	2nd-generation antihistamine	oral
Rupatadine Fumarate	2nd-generation antihistamine	oral
Mequitazine	2nd-generation antihistamine	oral
Tranilast	chemical mediator release inhibitor	oral
Pemirolast Potassium	chemical mediator release inhibitor	oral

Fexofenadine Hydrochloride + Pseudoephedrine Hydrochloride	2nd-generation antihistamine + α-stimulator	oral
Cortisone Acetate	steroid	oral
Hydrocortisone	steroid	oral
Dexamethasone	steroid	oral
Prednisolone	steroid	oral
Methylprednisolone	steroid	oral
Triamcinolone Acetonide	steroid	oral
Betamethasone	steroid	oral
Betamethasone + d-Chlorpheniramine Maleate	1st-generation antihistamine + steroid	oral
Pranlukast Hydrate	leukotriene receptor antagonist	oral
Montelukast Sodium	leukotriene receptor antagonist	oral
Ramatroban	prostaglandin D2 receptor/thromboxane A2 receptor antagonist	oral
Suplatast Tosilate	Th2 cytokine inhibitor	oral
Sodium Cromoglicate	chemical mediator release inhibitor	nasal drops
Ketotifen Fumarate	antihistamine	nasal drops
Levocabastine Hydrochloride	antihistamine	nasal drops

Betamethasone Acetate・Betamethasone Sodium Phosphate	steroid	injection
Betamethasone Sodium Phosphate	steroid	injection
Dexamethasone Sodium Phosphate	steroid	injection
Methylprednisolone Acetate	steroid	injection
Prednisolone Sodium Succinate	steroid	injection
Triamcinolone Acetonide	steroid	injection
Hydrocortisone Sodium Succinate	steroid	injection
Beclometasone Dipropionate	steroid	nasal drops
Fluticasone Propionate	steroid	nasal drops
Mometasone Furoate Hydrate	steroid	nasal drops
Fluticasone Furoate	steroid	nasal drops
Dexamethasone Cipecilate	steroid	nasal drops
Adrenaline	vasoconstrictor	nasal drops
Oxymetazoline Hydrochloride	vasoconstrictor	nasal drops
Tramazoline Hydrochloride	vasoconstrictor	nasal drops
Naphazoline Nitrate	vasoconstrictor	nasal drops
Tetrahydrozoline Hydrochlorid + Prednisolone	vasoconstrictor	nasal drops
Tyloxapol	expectorants	inhalants
Bromhexine Hydrochloride	respiratory organ agents	inhalants

Allergen (Ceder Pollen)	allergen immunotherapy	oral
Allergen Extracts (Cedar Pollen)	allergen immunotherapy	oral
Allergen Extracts (Japanese Red Pine Pollen)	allergen immunotherapy	oral
Allergen Extracts (Ragweed Pollen)	allergen immunotherapy	oral
Minor Blue Dragon Decoction	kampo	oral
d-Chlorpheniramine Maleate	antihistamine	injection
Chlorpheniramine Maleate	antihistamine	injection
Diphenhydramine Hydrochloride	antihistamine	injection
Diphenhydramine Hydrochloride・Calcium Bromide	antihistamine	injection
Emedastine Difumarate	antihistamine	patch
Omalizumab (Genetical Recombination)	anti-IgE antibody	injection
An extract from inflammatory rabbit skin inoculated by vaccinia virus	non-specific immunotherapy	injection
Histamine dihydrochloride and human normal immunoglobulin	non-specific immunotherapy	injection

NSAIDs, non-steroidal anti-inflammatory drugs.

Table S5. Treatment pattern for allergic conjunctivitis

	Group I	Group II	Group III	Group IV	Group V
Antiallergic ED	○	○	○	○	×
Steroid ED or NSAIDs ED	×	○	×	○	△
Antiallergic OM or PM	×	×	○	○	△

OM, oral medicine; ED, eye drops; NSAIDs, non-steroidal anti-inflammatory drugs; EO, eye ointments; PM, patch medicine.
If medications were prescribed, ○ was noted; if they were not prescribed, × was noted; otherwise, △ was noted.

Table S6. Treatment pattern for allergic rhinitis

	Group I: Mild	Group II: Moderate	Group III: Severe	Group IV: Other	Group V: None
Treatment	One of (a) to (f) or one of (b) to (e) + (f)	(c) or (d) + (f)+ (a) or (g) + (f)	Group II + (h) or (i) or (j) or (k) or much more	Other combinations of (a) to (k)	Not prescribed (a) to (k)

(a) 2nd-generation antihistamine oral medicines; (b) chemical mediator release inhibitor, oral medicine; (c) leukotriene receptor antagonist oral medicines; (d) prostaglandin D2 receptor/thromboxane A2 receptor antagonist oral medicines; (e) Th2 cytokine inhibitor oral medication; (f) nasal steroid drops; (g) 2nd-generation antihistamine and α stimulator combination oral medicines; (h) vasoconstrictor nasal drops; (i) steroid oral medicines; (j) steroid and 1st-generation antihistamine combination oral medicines; (k) anti-IgE antibody injection.

Table S7. Follow-up survey for regular outpatient visits among patients with HF before the COVID-19 pandemic

Patients	Patients with HF during the HF seasons before the COVID-19 pandemic		Patients with HF during the HF seasons after the COVID-19 pandemic	
Year	2018	2019	2020	2021
Number of patients with HF, individuals	1,084,620	1,084,620	746,932	719,657
Number of HF-related medications, individuals	4,977,468	5,027,852	3,356,985	3,325,623

HF, hay fever; COVID-19, coronavirus disease 2019.

References

1. Akasaki Y, Iwagami M, Sung J*, et al.* Impact of COVID-19 on care-seeking patterns for hay fever in Japan: A retrospective claims database cohort study. *Allergy* 2023.
